# Infective endocarditis according to type 2 diabetes mellitus status: an observational study in Spain, 2001–2015

**DOI:** 10.1186/s12933-019-0968-0

**Published:** 2019-11-21

**Authors:** José M. de Miguel-Yanes, Rodrigo Jiménez-García, Valentín Hernández-Barrera, Javier de Miguel-Díez, Manuel Méndez-Bailón, Nuria Muñoz-Rivas, Napoleón Pérez-Farinós, Ana López-de-Andrés

**Affiliations:** 1 Internal Medicine Department, Hospital General Universitario Gregorio Marañón, Universidad Complutense de Madrid, Instituto de Investigación Sanitaria Gregorio Marañón (IiSGM), 46, Doctor Esquerdo, 28007 Madrid, Spain; 2 Preventive Medicine and Public Health Teaching and Research Unit, School of Medicine, Complutense University, 58, Isaac Peral, 28040 Madrid, Spain; 3 Preventive Medicine and Public Health Teaching and Research Unit, Health Sciences Faculty, Rey Juan Carlos University, Avenida de Atenas s/n, 28922 Alcorcón, Madrid, Spain; 4 Respiratory Care Department, Hospital General Universitario Gregorio Marañón, Universidad Complutense de Madrid, Instituto de Investigación Sanitaria Gregorio Marañón (IiSGM), 46, Doctor Esquerdo, 28007 Madrid, Spain; 5 Internal Medicine Department, Hospital Universitario Clínico San Carlos, Universidad Complutense de Madrid, Profesor Martín Lagos, s/n, 28040 Madrid, Spain; 6 Internal Medicine Department, Hospital Universitario Infanta Leonor, 80, Avenida Gran Vía del Este, 28031 Madrid, Spain; 7 Public Health and Psychiatry Department, Faculty of Medicine, Universidad de Malaga, 32, Bulevar Louis Pasteur, 28071 Málaga, Spain

**Keywords:** Infective endocarditis, Type 2 diabetes mellitus, Heart valve surgery, Comorbidities, In-hospital mortality

## Abstract

**Background:**

The main aims of this study were to describe trends and outcomes during admission for infective endocarditis (IE) in people ≥ 40 years old with or without type 2 diabetes distributed in five time-periods (2001–2003; 2004–2006; 2007–2009; 2010–2012 and 2013–2015), using Spanish national hospital discharge data.

**Methods:**

We estimated admission rates by diabetes status. We analyzed comorbidity, therapeutic procedures, and outcomes. We built Poisson regression models to compare the adjusted time-trends in admission rates. Type 2 diabetes cases were matched with controls using propensity score matching (PSM). We tested in-hospital mortality (IHM) in logistic regression analyses.

**Results:**

We identified 16,626 hospitalizations in patients aged ≥ 40 years for IE in Spain, 2001–2015. The incidence of IE increased significantly from 6.0/100,000 per year to 13.1/100,000 per year (p < 0.001) in the population with type 2 diabetes, and from 3.9/100,000 per year to 5.5/100,000 per year (p < 0.001) in the population without diabetes, over the study period. The adjusted incidence of IE was 2.2-times higher among patients with diabetes than among those without diabetes (IRR = 2.2; 95% CI 2.1–2.3). People with type 2 diabetes less often underwent heart valve surgery than people without diabetes (13.9% vs. 17.3%; p < 0.001). Although IHM decreased significantly in both groups over time, it represented 20.8% of IE cases among diabetes patients and 19.9% among PSM matched controls (p = 0.337). Type 2 diabetes was not associated with a higher IHM in people admitted to the hospital for IE (OR = 1.1; 95% CI 0.9–1.2).

**Conclusion:**

Incidence rates of IE in Spain, among those with and without T2DM, have increased during the period 2001–2015 with significantly higher incidence rates in the T2DM population. In our population based study and after PSM we found that T2DM was not a predictor of IHM in IE.

## Background

The clinical profile of infective endocarditis (IE) has evolved during the last years: nowadays it is more often an acute disease diagnosed in an ageing population, with a high mortality rate [1, 2]. Research work from Spain has recently provided an overall view of the epidemiological and clinical characteristics of IE during the last years [3]. However, this report specifically focused on the changing patterns of microbiological isolations, and on evaluating the potential impact of differences among treating hospitals (i.e., hospital volume and number of IE cases) on patients’ outcomes.

Several reasons make necessary further investigations to assess the association between diabetes mellitus (DM) and IE; (i) Diabetes prevalence in increasing worldwide, and the proportion of diabetes among those suffering IE is rising in Spain and other countries [3–8]. As life expectancy increases, people including those suffering diabetes, will be exposed for a more extended period to predisposing factors for IE such as degenerative valvular lesions, prosthetic valves, intra-cardiac electronic devices or long-term intravenous lines [9]; (ii) The immunity is affected in patients with DM and as a consequence those suffering bacteremia are more prone to develop sepsis and IE [10–13]. Episodes of bacteremia occur more frequently in patients with DM, in part perhaps due to an increased rate of colonization of *Staphylococcus aureus* in the skin folds and nares of these individuals [12]. Further, patients with DM are prone to severe endothelial dysfunction, which is one of the central pathophysiologic steps in the development of IE [14, 15]; (iii) The number of epidemiological population-based studies that have suggested that DM increases the risk of IE is still limited [16–20], and (iv) A recent review to assess the predictors of in-hospital mortality (IHM) in patients with IE concluded that most studies are retrospective, provide data from one or few tertiary center, have long recruitment periods and sample sizes are usually small [21]. Regarding IHM after IE among diabetic patients the number of investigations is limited and results are inconsistent [13, 22–31].

Hence, using the Spanish National Hospital Discharge Database (SNHDD), in this study we aimed to: (i) examine the trends in the incidence, characteristics, and in-hospital outcomes of IE among patients with and without type 2 diabetes (T2DM) from 2001 to 2015; (ii) compare in-hospital outcomes for IE in patients with and without T2DM using PSM; and (iii) identify factors associated with IHM during admission for IE among patients with T2DM.

## Methods

### Data source and patient population

This retrospective observational study was performed using the SNHDD. Details of the design and description of the SNHDD are available online [32]. We received from the Spanish Ministry of Health the SNHDD including all patients with a primary (first position) or secondary diagnosis (positions 2 to 14) of IE (ICD-9-CM codes 421.0, 421.1, 421.9, 424.99) hospitalized from year 2001 to year 2015. These codes have been frequently used in previous studies of IE using hospital discharge databases [3, 4, 28, 33].

We defined as our study population all those aged ≥ 40 years with ICD9 codes for IE in the first and second position and excluding those with a ICD9 code for history of intravenous drug use (codes 304.0, 305.5) and HIV infection (codes 042.x–044.x). We confirmed that all cases included in our study sample did not have in the database another subject with a code for IE with the same age, sex, year of admission and hospital as this could be the same patient readmitted for an IE recurrence of an index admission. In these cases, as described by Olmos et al. [3], the first IE admission would be analyzed and the second admission discharged. Additional file 1: Figure S1 shows the Flow Chart of the patient selection. We grouped admissions by diabetes status as follows: T2DM (ICD-9-CM codes 250.x0 and 250.x2) or no diabetes in any diagnostic position. We excluded people with type 1 diabetes (codes 250.x1 and 250.x3).

### Covariates

Clinical characteristics included information on overall comorbidity at the time of discharge, which was assessed by calculating the Charlson Comorbidity Index (CCI) [34]. Calculation of the CCI was performed by excluding diabetes as a disease (therefore, we used a modified CCI). We retrieved data on prosthetic valve carriers and the following diagnoses reported for each episode: previous mitral valve disease, previous aortic valve disease, septic arterial embolism, ischemic heart disease, shock, and periannular complications/atrioventricular block. Regardless of the position in the procedure coding list, we captured data on the following in-hospital procedures: heart valve surgery, dialysis, pacemaker implantation and mechanical ventilation. We analyzed pathogens documented during hospitalizations for IE, including coagulase-negative Staphylococci, *S. aureus*, Streptococci, Enterococci, *Streptococcus pneumoniae*, anaerobes, Gram-negative bacteria and fungal infections (candidiasis/aspergillosis). The ICD-9-CM codes for the diagnoses identified and the procedures conducted during the hospitalization for IE that we included are shown in Additional file 2: Table S1.

We considered that a patient was a “Readmission” if that patient hospitalized with IE had been discharged from the same hospital in the previous 30 days without a diagnosis of IE. This variable is included in the SNHDD when we receive it from the Spanish Ministry of Health. In some way the variable “readmission” could be considered as a proxy of hospital-acquired EI.

The main end points were trends in incidence rates of hospitalizations and IHM, defined by the proportion of patients who died during admission for each year of study. We also estimated the mean of length of hospital stay (LOHS). The SNHDD does not include any information after the patient is discharged from the hospital. Therefore, no data were available on long-term outcomes. We calculated costs calculated using diagnosis-related groups for the disease [35] and analyzed them as inflation-adjusted.

### PSM method

T2DM status represents the treatment indicator introduced in the theoretical part of this study. As can be seen in Table 1, significant differences are found between people with or without T2DM for age and for almost every clinical variable analyzed. Therefore, it was deemed necessary to use a statistical method, such as PSM (PSM), to control the confounding effect of baseline characteristics. The main aim of the propensity score method is to obtain an unbiased estimate of treatment effect adjusted for the impact of given confounding factors in non-randomized and observational studies [36]. The PSM method allows selecting participants with and without T2DM with the same, or nearly the same propensity score obtained with logistic regression, so that we can match the structure of the confounding factors for both group of patients [36, 37]. By using multivariable logistic regression, we could estimate a propensity score for each person with and without T2DM in our investigation [36]. The variables included in the model were year of admission, sex, age, all comorbidities analyzed (Additional file 1: Table S1), previous aortic valve disease and prosthetic valve carriers.

**Table 1:** Incidence, clinical characteristics and in-hospital outcomes of patients hospitalized with infective endocarditis in Spain from 2001 to 2015 according to T2DM status

	T2DM	2001–2003	2004–2006	2007–2009	2010–2012	2013–2015	Total	Trend
Number of endocarditis	Yes	345	486	603	962	1040	3436	< 0.001
	No	2019	2128	2304	3165	3574	13,190	< 0.001
Incidence per 100,000 per year*	Yes	6.0	7.8	8.9	12.8	13.1	10.0	< 0.001
	No	3.9	3.8	3.8	5.1	5.5	4.5	< 0.001
Age, mean (SD)*	Yes	68.7 (9.8)	69.2 (9.9)	70.4 (9.9)	71.6 (10.2)	71.8 (10.3)	70.8 (10.2)	< 0.001
	No	66.2 (12.1)	66.0 (12.3)	67.4 (12.5)	69.0 (12.2)	69.4 (12.4)	67.9 (12.4)	< 0.001
Female sex, n (%)	Yes	130 (37.7)	193 (39.7)	233 (38.6)	348 (36.2)	336 (32.3)	1240 (36.1)	0.023
	No	667 (33.0)	704 (33.1)	786 (34.1)	1037 (32.8)	1131 (31.7)	4325 (32.8)	0.393
Charlson Comorbidity Index, mean (SD)*	Yes	1.0 (0.8)	1.0 (0.8)	1.0 (0.8)	1.1 (0.8)	1.1 (0.8)	1.1 (0.8)	< 0.001
	No	0.8 (0.8)	0.8 (0.8)	0.9 (0.8)	1.0 (0.8)	1.0 (0.8)	0.9 (0.8)	< 0.001
Prosthetic valve carriers, n (%)	Yes	27 (7.8)	41 (8.4)	57 (9.5)	106 (11.0)	102 (9.8)	333 (9.7)	0.375
	No	170 (8.4)	191 (9.0)	205 (8.9)	294 (9.3)	360 (10.1)	1220 (9.3)	0.280
Previous mitral valve disease, n (%)*	Yes	99 (28.7)	122 (25.1)	156 (25.9)	271 (28.2)	264 (25.4)	912 (26.5)	0.478
	No	540 (26.8)	616 (29.0)	681 (29.6)	1034 (32.7)	1100 (30.8)	3971 (30.1)	< 0.001
Previous aortic valve disease, n (%)*	Yes	75 (21.7)	114 (23.5)	149 (24.7)	251 (26.1)	280 (26.9)	869 (25.3)	0.280
	No	511 (25.3)	602 (28.3)	673 (29.2)	972 (30.7)	1122 (31.4)	3880 (29.4)	< 0.001
Readmissions, n (%)*	Yes	58 (16.8)	91 (18.7)	116 (19.2)	199 (20.7)	223 (21.4)	687 (20.0)	0.338
	No	300 (14.9)	314 (14.8)	354 (15.4)	526 (16.6)	597 (16.7)	2091 (15.9)	0.128
Length of hospital stay, mean (SD)	Yes	28.24 (20.9)	28.9 (21.4)	28.48 (20.4)	27.69 (21.0)	24.76 (18.9)	27.17 (20.4)	< 0.001
	No	28.33 (22.6)	27.87 (21.8)	28.25 (22.4)	27.18 (22.4)	26.66 (21.4)	27.51 (22.1)	0.018
In-hospital mortality, n (%)	Yes	87 (25.2)	101 (20.8)	135 (22.4)	209 (21.7)	182 (17.5)	714 (20.8)	0.015
	No	431 (21.4)	397 (18.7)	439 (19.1)	638 (20.2)	657 (18.4)	2562 (19.4)	0.053
Costs, mean (SD)*	Yes	9137.6 (5486)	12,380.7 (8116.2)	14,928.4 (8646.3)	13,507.4 (11,465.3)	13,744.5 (10,822.8)	13,230.4 (10,000.7)	< 0.001
	No	10,171.7 (7450.2)	12,746.6 (8463.6)	16,366.4 (11,276.8)	16,588.5 (17,974.3)	14,759.3 (12,184.5)	14,452.0 (12,845.6)	< 0.001

*T2DM* type 2 diabetes mellitus, *SD* standard deviation

*Denotes a p value < 0.05 for the difference when comparing total values between patients with and without T2DM (namely, the results in the “TOTAL” column). Trends for incidence were assessed using multivariable Poisson regression models adjusted by age and sex

For PSM we used the PSMATCH2 Stata module. The Matching method chosen was one-to-one within caliper of width equal to 0.2 of the standard deviation of the logit of the propensity score [38, 39]. Using this method, we could match all case of T2DM with a non-diabetic patient. To assess the balance of the samples after PSM we estimated the absolute standardized difference before and after matching. As can be seen in Additional file 3: Table S2 none of the absolute standardized differences after PSM were above 10%, that represents meaningful imbalance.

### Statistical analysis

We considered five time-periods that included 3 consecutive years each (2001–2003; 2004–2006; 2007–2009; 2010–2012 and 2013–2015). To assess time trends, we estimated the incidence rates of admission for IE among people with and without T2DM calculated per 100,000 individuals per year We calculated T2DM-specific incidence rates by dividing the number of admissions per year, sex, and age group by the corresponding number of people in that population group using the age-adjusted, sex-adjusted estimated prevalence of T2DM obtained from National Health Surveys (NHS) conducted in 2001/02, 2003/04, 2006/07, 2009/10, 2011/12, and 2014/15, and based on data from the Diabetes Study, which estimated the prevalence of diabetes in the Spanish population [5, 40]. Data for the diabetic population for missing years (2005, 2008 and 2013) was estimated assuming that the growth rate was the same through the period 2004–2014. We estimated rates by fitting a linear regression model with population from years when NHS was available, and we used this model to impute population data for 2005, 2008, and 2013. We also calculated incidence rates for non-diabetic patients by dividing the number of cases per year, sex, and age group by the corresponding number of people in that population group (excluding those with T2DM), according to the data from the Spanish National Institute of Statistics, as reported on 31 December of each year [41].

A descriptive statistical analysis was performed for all continuous and categorical variables. Variables are expressed as means with standard deviations and as proportions. A bivariable analysis according to year was performed using the χ^2^ test for linear trend and ANOVA, as appropriate. To assess differences between those patients with and without T2DM, for each year and for the total sample, the statistical tests conducted for continuous variables were the T test for normal distributions and the Mann–Whitney test for non-normal distributions; categorical variables were compared using the Chi-square test. Incidence rates were compared using multivariable Poisson regression adjusted by age and sex. Estimates correspond to Incidence Rate Ratios (IRRs), with their 95% confidence intervals (95% CIs).

The Paired t-test was used for continuous variables and the McNemar’s test was used for categorical variables to compare people with and without T2DM after PSM. To identify variables associated with IHM as a binary outcome among patients with IE, we performed three multivariable logistic regression analyses (T2DM, no diabetes, and both). The variables included in the models were those with significant results in the bivariable analysis plus those considered relevant in other investigations. The multivariable model was conducted in the PSM samples. Estimates were the odds ratios (ORs), with their 95% CIs.

The multivariable models were built using the “enter modelling” method of STATA 14.0 The process included the following steps:

1. Each independent variable was analyzed according to diabetes status, (bivariate analysis).
2. Selection of variables for the multivariable analysis. We included those significant variables in the bivariate analysis and those found scientifically relevant by other authors.
3. To fit the multivariable model we used the Wald statistic (WS) to assess the importance of each variable. Those variables that according to this test we considered that did not improve the model were deleted, and a new model was re-analyzed. (expert opinion method for independent variable selection). Likelihood ratio test was used to compare successive models with previous ones.
4. Once the final model was fitted, we checked for collinearity and interactions between the remaining variables.

All statistical analysis was performed with Stata version 14.0 (Stata, College Station, Texas, USA). Statistical significance was set at p < 0.05 (2-tailed).

### Ethical aspects

Data confidentiality was maintained all the time. Patient identifiers were deleted before the database was provided to the authors, to keep patient anonymity. It is not possible to identify patients on an individual level, either in this article or in the database. Given the anonymous and mandatory nature of the dataset, the Ethics Committee of the Rey Juan Carlos University in Madrid informed that it was not necessary to obtain any kind of additional informed consent, in accordance with the Spanish legislation.

## Results

### Overall incidence of infective endocarditis and according to T2DM status

We identified a total of 16,626 hospitalizations of patients aged 40 years or older with a diagnosis of IE in Spain (2001–2015). Patients with T2DM accounted for 20.7% of the total (1240 women and 2196 men).

Among patients with T2DM, we found that the incidence of IE coding increased significantly from 6.0 in 2001–2003 to 13.1 cases per 100,000 T2DM population per year in 2013–2015 (p < 0.001). In patients without T2DM the incidence of admissions also increased significantly over the study period from 3.9 to 5.5 cases per 100,000 non-T2DM population per year (p < 0.001) (Table 1). Incidence was significantly higher in people with T2DM than in non-diabetic people for all years analyzed (p < 0.05). Using the Poisson regression model, adjusting by age and sex, we found that the incidence of IE was 2.2-times higher among patients with T2DM than among those without diabetes (IRR 2.2; 95% CI 2.1–2.3).

### Clinical characteristics and in-hospital outcomes of patients hospitalized with infective endocarditis according to T2DM status

In patients who had an admission for IE there was a significant male predominance (63.9% in T2DM and 67.31% in the no-diabetes population). Overall, patients with T2DM were older (70.8 ± 10.2 years) than patients without diabetes (67.9 ± 12.4 years) and had more coexisting medical conditions (mean modified CCI index 1.1 ± 0.8 vs. 0.9 ± 0.8) (all p values < 0.001). In contrast, previous mitral and aortic valve disease were more prevalent in people without diabetes, as can been seen in Table 1.

Readmission rates were significantly higher in patients with T2DM (20.0% vs. 15.9%). Mean LOHS was around 27 days in both groups and the mean cost per patient was significantly lower in people with T2DM (13,230.4 euros vs. 14,452.0 euros). Over time, LOHS fell significantly in both groups of patients and costs increased significantly.

For the total time period, crude IHM was 20.8% for people with T2DM and 19.4% for people without diabetes (p = 0.075). IHM tended to decrease over time in both groups, though statistical significance was only reached among people with T2DM (from 25.2% in 2001–2003 to 17.5% in 2013–2015; p = 0.015) (Table 1).

Shown in Additional file 4: Table S3 and Additional file 5: Table S4 are the baseline conditions by study periods among those patients suffering IE with and without concomitant T2DM.

### Distribution of study covariates and IHM among patients with and without T2DM hospitalized with infective endocarditis in Spain

Table 2 shows the distribution of the study variables and Table 3 shows IHM according to these variables among people with and without T2DM admitted for IE, before and after PSM. When we compared patients with T2DM with matched controls after PSM, we still found significantly lower rates of heart valve surgery in the people with T2DM (13.9% and 17.3%, respectively; p < 0.001) (Table 2). However, dialysis was significantly more prevalent in patients with T2DM than in matched controls (7.2% vs. 5.9%, p = 0.028).

**Table 2:** Distribution of study covariates and hospital outcomes of patients with and without T2DM hospitalized with infective endocarditis in Spain from 2001 to 2015, before and after propensity score matching

	Before matching			After matching		
	T2DM	Non T2DM	p value	T2DM	Non T2DM	p value
Time period, n (%)						
2001–2003	345 (10.0)	2019 (15.3)	< 0.001	345 (10.0)	371 (10.8)	0.277
2004–2006	486 (14.1)	2128 (16.1)		486 (14.1)	456 (13.3)	
2007–2009	603 (17.6)	2304 (17.5)		603 (17.6)	576 (16.8)	
2010–2012	962 (28.0)	3165 (24.0)		962 (28.0)	928 (27.0)	
2013–2015	1040 (30.3)	3574 (27.1)		1040 (30.3)	1105 (32.2)	
Total	3436 (100)	13,190 (100)		3436 (100)	3436 (100)	
Sex, n (%)						
Male	2196 (63.9)	8865 (67.2)	< 0.001	2196 (63.9)	2175 (63.3)	0.599
Female	1240 (36.1)	4325 (32.8)		1240 (36.1)	1261 (36.7)	
Age, mean (SD)	70.8 (10.2)	67.9 (12.4)	< 0.001	70.8 (10.2)	70.3 (11.8)	0.154
Age groups, n (%)						
40–66 years old	1030 (30.0)	5437 (41.2)	< 0.001	1030 (30.0)	1067 (31.1)	0.001
67–75 years old	1153 (33.6)	3547 (26.9)		1153 (33.6)	1014 (29.5)	
≥ 76 years old	1253 (36.5)	4206 (31.9)		1253 (36.5)	1355 (39.4)	
CCI, mean (SD)	1.1 (0.8)	0.9 (0.8)	< 0.001	1.1 (0.8)	1.1 (0.8)	0.673
Prosthetic valve carriers, n (%)	333 (9.7)	1220 (9.3)	0.428	333 (9.7)	306 (8.9)	0.262
Previous mitral valve disease, n (%)	912 (26.5)	3971 (30.1)	< 0.001	912 (26.5)	908 (26.4)	0.913
Previous aortic valve disease, n (%)	869 (25.3)	3880 (29.4)	< 0.001	869 (25.3)	846 (24.6)	0.521
Congestive heart failure, n (%)	972 (28.3)	3666 (27.8)	0.565	972 (28.3)	935 (27.2)	0.319
Septic arterial embolism, n (%)	39 (1.1)	164 (1.2)	0.607	39 (1.1)	46 (1.3)	0.445
Dementia, n (%)	61 (1.8)	153 (1.2)	0.004	61 (1.8)	56 (1.6)	0.641
Acute renal disease, n (%)	617 (18.0)	2432 (18.4)	0.516	617 (18.0)	598 (17.4)	0.548
Chronic renal disease, n (%)	592 (17.2)	1329 (10.1)	< 0.001	592 (17.2)	536 (15.6)	0.068
Ischemic heart disease, n (%)	627 (18.3)	1467 (11.1)	< 0.001	627 (18.3)	611 (17.8)	0.616
COPD, n (%)	602 (17.5)	2167 (16.4)	0.126	602 (17.5)	643 (18.7)	0.199
Atrial fibrillation, n (%)	863 (25.1)	2916 (22.1)	< 0.001	863 (25.1)	867 (25.2)	0.911
Shock, n (%)	239 (7.0)	1099 (8.3)	0.008	239 (7.0)	221 (6.4)	0.385
Periannular complications/atrioventricular block, n (%)	129 (3.8)	600 (4.6)	0.043	129 (3.8)	165 (4.8)	0.032
Heart valve surgery, n (%)	479 (13.9)	2630 (19.9)	< 0.001	479 (13.9)	595 (17.3)	< 0.001
Dialysis, n (%)	248 (7.2)	761 (5.8)	< 0.001	248 (7.2)	203 (5.9)	0.028
Pacemaker implantation, n (%)	89 (2.6)	356 (2.7)	0.725	89 (2.6)	86 (2.5)	0.818
Mechanical ventilation, n (%)	410 (11.9)	1774 (13.5)	0.019	410 (11.9)	412 (12.0)	0.941
Coagulase-negative staphylococci, n (%)	441 (12.8)	1463 (11.1)	0.004	441 (12.8)	399 (11.6)	0.122
*Staphylococcus aureus,* n (%)	504 (14.7)	1685 (12.8)	0.003	504 (14.7)	452 (13.2)	0.070
Streptococci, n (%)	649 (18.9)	2897 (22.0)	< 0.001	649 (18.9)	749 (21.8)	0.003
Enterococci, n (%)	557 (16.2)	1887 (14.3)	0.005	557 (16.2)	488 (14.2)	0.020
*Streptococcus pneumoniae,* n (%)	15 (0.4)	58 (0.4)	0.980	15 (0.4)	13 (0.4)	0.705
Anaerobes, n (%)	21 (0.6)	71 (0.5)	0.608	21 (0.6)	19 (0.6)	0.751
Gram-negative bacilli, n (%)	264 (7.7)	1012 (7.7)	0.983	264 (7.7)	260 (7.6)	0.856
Candidiasis/aspergillosis, n (%)	3 (0.1)	32 (0.2)	0.077	3 (0.1)	11 (0.3)	0.032
Readmissions, n (%)	687 (20.0)	2091 (15.9)	< 0.001	687 (20.0)	632 (18.4)	0.092
Length of hospital stay, mean (SD)	27.17 (20.4)	27.51 (22.1)	0.405	27.17 (20.4)	27.43 (22.1)	0.616
In-hospital mortality, n (%)	714 (20.8)	2562 (19.4)	0.075	714 (20.8)	682 (19.9)	0.337
Cost, mean (SD)	13,230.4 (10,000.7)	14,452.0 (12,845.6)	< 0.001	13,230.4 (10,000.7)	14,045.3 (12,713.2)	0.003

*T2DM* type 2 diabetes mellitus, *SD* standard deviation, *CCI* Charlson Comorbidity Index, *COPD* chronic obstructive pulmonary disease

**Table 3:** In-hospital mortality of patients with and without T2DM hospitalized with infective endocarditis in Spain from 2001 to 2015 according to study covariates, and hospital outcomes before and after propensity score matching

	In-hospital mortality before matching			In-hospital mortality after matching		
	T2DM	Non T2DM	p value	T2DM	Non T2DM	p value
Time period, n (%)						
2001–2003	87 (25.2)	431 (21.4)	0.109	87 (25.2)	95 (25.6)	0.995
2004–2006	101 (20.8)	397 (18.7)	0.282	101 (20.8)	92 (20.2)	0.818
2007–2009	135 (22.4)	439 (19.1)	0.067	135 (22.4)	112 (19.4)	0.215
2010–2012	209 (21.7)	638 (20.2)	0.292	209 (21.7)	194 (20.9)	0.663
2013–2015	182 (17.5)	657 (18.4)	0.516	182 (17.5)	189 (17.1)	0.809
Sex, n (%)						
Male	414 (18.9)	1592 (18.0)	0.33	414 (18.9)	405 (18.6)	0.844
Female	300 (24.2)	970 (22.4)	0.192	300 (24.2)	277 (22.0)	0.186
Age, mean (SD)	73.2 (9.7)	71.5 (11.4)	< 0.001	73.2 (9.7)	72.9 (10.9)	0.540
Age groups, n (%)						
40–66 years old	150 (14.6)	754 (13.9)	0.555	150 (14.6)	154 (14.4)	0.933
67–75 years old	228 (19.8)	696 (19.6)	0.91	228 (19.8)	194 (19.1)	0.706
≥ 76 years old	336 (26.8)	1112 (26.4)	0.791	336 (26.8)	334 (24.7)	0.206
CCI, mean (SD)	1.3 (0.7)	1.2 (0.8)	0.001	1.3 (0.7)	1.3 (0.7)	0.484
Prosthetic valve carriers, n (%)	57 (17.1)	213 (17.5)	0.884	57 (17.1)	56 (18.3)	0.695
Previous mitral valve disease, n (%)	190 (20.8)	723 (18.2)	0.067	190 (20.8)	180 (19.8)	0.593
Previous aortic valve disease, n (%)	184 (21.2)	730 (18.8)	0.111	184 (21.2)	161 (19.0)	0.269
Congestive heart failure, n (%)	274 (28.2)	1108 (30.2)	0.218	274 (28.2)	284 (30.4)	0.295
Septic arterial embolism, n (%)	9 (23.1)	45 (27.4)	0.58	9 (23.1)	10 (21.7)	0.883
Dementia, n (%)	18 (29.5)	46 (30.1)	0.936	18 (29.5)	16 (28.6)	0.911
Acute renal disease, n (%)	241 (39.1)	914 (37.6)	0.499	241 (39.1)	224 (37.5)	0.566
Chronic renal disease, n (%)	161 (27.2)	354 (26.6)	0.798	161 (27.2)	135 (25.2)	0.444
Ischemic heart disease, n (%)	167 (26.6)	338 (23.0)	0.078	167 (26.6)	143 (23.4)	0.190
COPD, n (%)	129 (21.4)	463 (21.4)	0.974	129 (21.4)	138 (21.5)	0.989
Atrial fibrillation, n (%)	176 (20.4)	662 (22.7)	0.152	176 (20.4)	201 (23.2)	0.160
Shock, n (%)	169 (70.7)	727 (66.2)	0.175	169 (70.7)	157 (71.0)	0.938
Periannular complications/atrioventricular block, n (%)	31 (24.0)	148 (24.7)	0.879	31 (24.0)	38 (23.0)	0.841
Heart valve surgery, n (%)	108 (22.6)	622 (23.7)	0.6	108 (22.6)	149 (25.0)	0.341
Dialysis, n (%)	115 (46.4)	409 (53.8)	0.044	115 (46.4)	94 (46.3)	0.989
Pacemaker implantation, n (%)	16 (18.0)	51 (14.3)	0.39	16 (18.0)	11 (12.8)	0.344
Mechanical ventilation, n (%)	204 (49.8)	883 (49.8)	0.995	204 (49.8)	213 (51.7)	0.577
Coagulase-negative staphylococci, n (%)	88 (20.0)	303 (20.7)	0.73	88 (20.0)	79 (19.8)	0.955
*Staphylococcus aureus,* n (%)	142 (28.2)	476 (28.3)	0.974	142 (28.2)	125 (27.7)	0.858
Streptococci, n (%)	81 (12.5)	276 (9.5)	0.024	81 (12.5)	76 (10.2)	0.169
Enterococci, n (%)	96 (17.2)	285 (15.1)	0.223	96 (17.2)	72 (14.8)	0.276
*Streptococcus pneumoniae,* n (%)	1 (6.7)	9 (15.5)	0.389	1 (6.7)	2 (15.4)	0.469
Anaerobes, n (%)	2 (9.5)	9 (12.7)	0.697	2 (9.5)	3 (15.8)	0.555
Gram-negative bacilli, n (%)	50 (18.9)	226 (22.3)	0.234	50 (18.9)	51 (19.6)	0.845
Candidiasis/aspergillosis, n (%)	2 (66.7)	14 (43.8)	0.459	2 (66.7)	6 (54.6)	0.708
Readmissions, n (%)	152 (22.1)	477 (22.8)	0.709	152 (22.1)	144 (22.8)	0.774
Length of hospital stay, mean (SD)	19.8 (19.2)	22.8 (23.9)	0.002	19.8 (19.2)	23.5 (24.2)	0.002
Cost, mean (SD)	14,856.9 (12,956.0)	18,334.8 (18,939.4)	< 0.001	14,856.9 (12,956.0)	17,408.9 (18,431.4)	0.003

*T2DM* type 2 diabetes mellitus, *SD* standard deviation, *CCI* Charlson Comorbidity Index, *COPD* chronic obstructive pulmonary disease

In our study sample, and before matching, the prevalence of *S. aureus* (14.7% vs. 12.8% p = 0.003) and Enterococci (16.2% vs. 14.3%; p < 0.001) were higher among T2DM patients whereas Streptococci (18.9% vs. 22.0%; 0.005) was more frequently identified among those without T2DM. However, when PSM was conducted the difference between both groups in the prevalence of *S. aureus* became not significant (Table 3).

Among T2DM patients 130 (3.78%) had two pathogens coded and 4 (0.12%) had three. The most frequent combinations were Gram-negative bacilli + *S. aureus* (n = 41, 31.54%), Gram-negative bacilli + Enterococci n (n = 33, 25.38%) and Gram-negative bacilli + Streptococci (n = 26, 20%). Among those without diabetes 486 (3.68%) had two and 33 (0.25%) three. The combinations of two pathogens were Gram-negative bacilli + *S. aureus* (n = 179, 36.83%), Gram-negative bacilli + Enterococci (n = 105, 21.61%) and Gram-negative bacilli + Streptococci (n = 97, 19.96%).

Overall IHM during admission for IE was 20.8% in participants with T2DM and 19.9% in matched controls (p = 0.337). Specifically focusing on people who died during admission for IE, the mean LOHS were 19.8 ± 19.2 days among people with T2DM and 23.5 ± 24.2 days among matched controls (p = 0.002) (Table 3), and the mean cost per patient was 14,856.9 euros among patients with T2DM and 17,408.9 euros among matched controls (p = 0.003).

### Multivariable logistic regression analysis of the factors associated with IHM

Additional file 6: Table S5 shows which study variables were associated with the IHM in the bivariate analysis among those with and without T2DM. All those variables significantly associated with IHM were included in the multivariable model. The variables not always significant and included in the multivariable models because they were considered relevant in other investigations were: prosthetic valve carriers, previous mitral valve disease, previous aortic valve disease, COPD, Gram-negative bacilli, candidiasis/aspergillosis, readmissions, heart valve surgery, Coagulase-negative staphylococci and *Streptococcus pneumoniae*. Table 4 shows the result of the multivariable logistic regression analysis of the factors independently associated with IHM according to the presence of T2DM. Over time, IHM decreased significantly regardless of the presence of T2DM. Female sex was a significant risk factor for IHM in people with T2DM (OR 1.3; 95% CI 1.1–1.6). Among people with T2DM admitted for IE, IHM was significantly higher in older patients (OR 1.8; 95% CI 1.6–2.0), in patients with more comorbidities according to the CCI (OR 1.6; 95% CI 1.5–1.8). Lastly, in our study, after adjusting for all the study—not only basal—variables, and supporting what we found in the PSM model, T2DM was not associated with a higher IHM in people admitted to the hospital with an episode of IE (OR, 1.1; 95% CI 0.9–1.2).

**Table 4:** Multivariable analysis of factors associated with in-hospital mortality during admission for infective endocarditis according to T2DM status

	T2DM	No T2DM	Total
	OR (95% CI)	OR (95% CI)	OR (95% CI)
Time period	0.8 (0.8–0.9)	0.8 (0.8–0.9)	0.8 (0.8–0.9)
Female sex	1.3 (1.1–1.6)	NS	1.2 (1.0–1.3)
Age^a^	1.8 (1.6–2.0)	1.8 (1.6–2.0)	1.8 (1.7–2.0)
Charlson Comorbidity Index	1.6 (1.5–1.8)	1.6 (1.5–1.8)	1.7 (1.5–1.8)
Acute renal disease	2.0 (1.6–2.5)	1.9 (1.5–2.4)	2.0 (1.7–2.3)
Ischemic heart disease	1.3 (1.0–1.6)	NS	NS
COPD	0.7 (0.5–0.9)	0.7 (0.5–0.9)	0.7 (0.6–0.8)
Shock	8.6 (6.1–12.1)	7.1 (5.0–10.1)	7.4 (5.8–9.5)
Dialysis	2.5 (1.8–3.5)	1.8 (1.3–2.6)	2.1 (1.7–2.7)
Pacemaker implantation	NS	0.4 (0.2–0.8)	0.5 (0.3–0.9)
Mechanical ventilation	3.3 (2.5–4.4)	3.7 (2.8–4.8)	3.4 (2.8–4.1)
*Staphylococcus aureus*	NS	NS	1.2 (1.0–1.5)
Streptococci	0.6 (0.5–0.8)	0.4 (0.3–0.6)	0.5 (0.4–0.6)
Enterococci	NS	0.6 (0.5–0.9)	0.8 (0.6–0.9)
*Streptococcus pneumoniae*	NS	NS	0.2 (0.0–1.0)
T2DM	NA	NA	1.1 (0.9–1.2)

Only those variables that showed a significant association after multivariable adjustment are showed

*T2DM* type 2 diabetes mellitus, *COPD* chronic obstructive pulmonary disease, *OR* odds ratio obtained using logistic regression models, *95% CI* 95% confidence intervals, *NS* not significant, *NA* Not applicable

^a^Age was introduced as a continuous variable with three categories. Additional file 6: Table S5 shows which study variables were associated with the IHM in the bivariate analysis among those with and without T2DM. All those variables significantly associated with IHM were included in the multivariable model. The variables not always significant and included in the multivariable models because they were considered relevant in other investigations were: prosthetic valve carriers, previous mitral valve disease, previous aortic valve disease, COPD, Gram-negative bacilli, Candidiasis/aspergillosis, readmissions, heart valve surgery, Coagulase-negative staphylococci and *Streptococcus pneumoniae*

## Discussion

Here we found increasing incidence rates for IE in the Spanish population during the period 2001–2015, which were over twofold higher among the people with T2DM than among their non-diabetic counterparts. Over time, mean LOHS was around 27 days, and fell significantly in both people with and without T2DM, whereas costs increased significantly. IHM was around 20% both in the people with and without T2DM. Finally, in our study T2DM was not associated with a higher IHM during admission for IE.

Other authors have also identified increasing incidence rates for IE [19]. The burden of comorbidity of an ageing population, a progressively higher number of invasive procedures, and other factors like current trends to prescribe antibiotics to prevent IE less often have been underscored by these researchers as possible reasons. However, the results from these publications are difficult to reconcile with other studies that have not detected increasing incidence rates for IE [4]. T2DM has been proposed to induce endothelial dysfunction, which can promote bacterial adhesion and in consequence predispose patients to the onset of IE [14, 15]. Additional mechanisms that could potentially contribute to incident IE in T2DM are the impaired immune response reported in older studies among people with diabetes [10], or the higher prevalence of certain types of infection among the people with diabetes that are known to generate bacteremias [12].

We found that *S. aureus* and Enterococci were more frequent among T2DM patients and Streptococci among those without T2DM. The higher incidence of *S. aureus* infection among patients with DM and IE is consistent with previous studies [12, 23, 24]. Two probable causes suggested for this are that DM patients use healthcare services more frequently and thus have a higher chance of being exposed [6, 8, 17] and that diabetic patients have relatively higher risk for skin and mucous membrane bacterial infection compared to nondiabetic patients [12, 19, 23]. Lin et al. found a higher rate of *S. aureus*-related IE in patients with DM (41.7% in patients with DM vs. 27.9% in patients without DM; p = 0.021). The frequencies of other microorganism-related IEs were not different between the two groups [24]. Kourany et al. compared microorganisms among diabetic and nondiabetic patients suffering IE and reported that *S. aureus* was isolated more often (30.7% vs 21.7%, p = 0.02), and from the viridans Streptococcus group less often (16.7% vs 28.2%, p  =  0.001) in the diabetic group [13]. Olmos et al. [23] described that enterococcus and *S. bovis* were more frequent in patients with DM, whereas the viridans group streptococci were more commonly isolated in those without. Chirillo et al. [12] analyzed 309 episodes of IE, of whom 70 had DM and Enterococcus was more commonly isolated in this last group.

Higher valve surgery rate in patients without DM before (13.9% vs 19.9%) and after matching (13.9% vs 17.3%) was found in our study. However as can be seen in Additional file 6: Table S5 the IHM did not differ between those T2DM patients who underwent or not valve surgery (22.6% vs. 20.49% respectively; p = 0.304). Chu et al. [25], in a prospective cohort of consecutively enrolled patients with definitive IE from 29 centers in 16 countries found that after multivariable analysis suffering DM was a negative predictor for surgical treatment for IE (OR = 0.52 [0.39, 0.69]). Other authors agree with us finding a lower surgical intervention rate in patients with DM [24].

In our investigation the mean length of hospital stay was around 27 days for those with and without T2DM. These values are much higher than those reported in investigations conducted in US [4, 33]. Recently Morita et al. describe a median length of stay of 10 (IQR, 6–17) days for those who survived to hospital discharge after IE during the period 2010–2014. Our equivalent figures for those who survived were over 28 days. From 1999 to 2010, using the Medicare inpatient Standard Analytic Files, Bikdeli et al. [4] reported that the mean length of stay for hospitalizations for endocarditis among patients aged 65 years of over consistently declined, from 9.6 (SD 11.5) days to 8.4 (SD: 8.9) days (p < 0.001). However they reported that around one quarter of patients was discharged to an intermediate care facility/skilled nursing facility, and another 10–17% to home health care. In Spain most patients (over 80%) are discharged to their home without any healthcare assistance and this may partly explain why the stay at the hospital is longer in our country. A report from Italy showed that median length of stay excluding hospital transfers increased from 23 to 25 days from 2000 to 2008, when hospital transfers were included the figures were 30 and 35 days respectively [28]. In Denmark the median admission length was 31 days (25 and 75 percentiles: 13–45 days) [17]. These last two countries have similar health service organization than Spain. Finally, in Brazil over a total of 203 consecutive patients admitted to a single tertiary care hospital between September 2005 and April 2017 the median duration of hospital stay was 42 days, ranging from 1 to 179 days [30].

Length of hospital stay is high for this condition, albeit it has been reduced somewhat in recent years. Perhaps, a faster identification of bacteremia with novel methods, the urgent notification of these positive microbiological results, a greater awareness on behalf of clinicians to proceed with an early transesophageal echocardiography at the detection of certain types of bacteremia [42], may be responsible for the observed reduction in the LOHS. Newly established strategies, such as sequential oral treatment for IE due to several types of microorganisms in patients with uncomplicated courses will probably allow even shorter regimens of intravenous treatment and lower hospital stays [43].

It is worth pointing out that mean cost per patient was consistently lower in people with T2DM than in people without diabetes. We do not have a straight explanation for this. The lower rates of heart valve surgery in the people with T2DM [24, 25, 44], or significantly shorter LOHS among the people with T2DM who died may partially account for this finding, but these arguments are merely speculative, since we lack the tools needed to provide a convincing reason to support this finding.

We were not surprised to find that hospital admission for IE had an overall high mortality (≈ 20%), although IHM decreased significantly over time. This figure is very similar to the rates described in the literature [45]. Except for occasional reports [46], most studies have also shown improving outcomes in recent years.

In our opinion the larger decrease in the crude IHM found among those with T2DM than among non-diabetic patients (Table 1) may be a consequence of the improvement in the management and pharmacological treatment of T2DM patients that has been described in Spain over the last years [47].

We found female sex to be an independent risk factor for IHM in people with T2DM admitted for IE. In our environment, previous research has linked this association to gender-related differences in the rates of heart valve surgery [48], but rates of surgery did not differ between men and women in our study (data not shown). Other authors have claimed that the differences between genders regarding outcomes in IE most likely result from dissimilarities in coexisting medical conditions [49]. Notwithstanding the importance of this topic, we cannot give a rational explanation for this finding.

After multivariable analysis valve surgery was not associated with IHM beside diabetes status. A previous review has reported that early valve surgery improve the outcome of patients with IE [50]. Unfortunately, with our database it is not possible to know the time until surgery was conducted so the existence of a bias with this variable cannot be discarded.

T2DM was not associated with a higher IHM in our multivariable analysis. In the literature, both opposite and similar results have been published [13, 22–31].

Lin et al. [24] in a single tertiary care hospital showed that after adjusting the other confounding factors patients with DM had 3.29 times greater IHM rate compared to patients without DM. Kourany et al. [13] found similar results after multivariable analysis of 1055 people with IE (150 with DM) where DM was an independent predictor of mortality (OR 1.71, 95% CI 1.08–2.70), with crude IHM figures of 30.3% vs 18.6%, p  =  0.001).

Some reasons have been suggested for the worse outcome in DM patients including: longer time from admission to the diagnosis of IE in the DM population which would resulted in the delay of suitable antibiotics treatment of and/or surgical intervention and the greater prevalence of *S. aureus* infection that is associated with higher rates of both complications and mortality in IE [13, 22, 24, 25].

However other studies didn’t identify DM as a risk factor [9, 23, 26, 28–31]. Fedeli et al. and Sy et al. in population based studies conducted in Veneto Region (North-Eastern Italy) and Australia did not find that DM was a risk factor for mortality [9, 28]. A previous Spanish investigation based in three tertiary care centers reported that multivariable analysis showed that DM had an independent association with development of septic shock (OR 2.282; 95% CI 1.186–4.393), but it was not a predictor of IHM [23]. In France among 4405 IE patients admitted to ICU, 14% suffering DM, this condition was not a predictor of IHM [31].

Unfortunately, data on glycemic control (glucose levels, glycated hemoglobin) are not available in our database, and we cannot evaluate their impact on patient outcome. The only possible way to assess glycemic control in the SHDD is using ICD-9-CM codes 250.x0 and 250.x2, indicating controlled DM and uncontrolled DM, respectively. Doing this we found that 3320 (96.62%) have 250.x0 and only 116 (3.38%) have 250.x2 with the IHM being similar for both groups (688/3320; 20.72% vs. 26/116; 22.41%; p = 0.66). The extremely low prevalence of code 250.x2 makes in our opinion this method not useful to assess glycemic control.

It is known that the strict glycemic control can improve the cellular immunity and as a consequence the incidence and clinical prognosis of patients with DM when affected by critical cardiovascular illness [11, 18, 22, 24]. Critchley et al. describe that the largest relative associations between the poorest level of glycemic control (HbA1c < 11%) and optimal control (6–7%) were seen for bone and joint infections (IRR 8.71), endocarditis (IRR 5.56), and sepsis (IRR 3.64). However, the highest attributable risk fraction, defined as the percentage of infections that would not have occurred if all individuals had the same infection risk as those in the optimal control group of HbA1c (6–7%), was observed for endocarditis (26.2%) [18].

Wei et al. compared the IHM for three groups of patients (normoglycaemia, prediabetes and diabetes, according to the American Diabetes Association) and suffering IE: The IHM found were 3.4%, for normoglycaemia patients, 12.6% for those with prediabetes and 17.9%, for the diabetes groups (p < 0.001). Compared with the normoglycaemia group, the adjusted OR for IHM was 2.42 (95% CI 1.11–5.31) for prediabetes and 3.39 (95% CI 1.48–7.80) for the diabetes group [22]. A larger, prospective, and more detailed (DM therapy, glycemic control, etc.) study is needed.

The strength and the novelty of our investigation are justified by the following reasons. First, we have analyzed data from an entire country over a 15-year period, providing data from 3436 T2DM patients suffering IE, this is one of the largest sample size ever described. Secondly, we use a PSM to improve the analysis of data, to our knowledge this method has not been used before for this topic. Finally, we focus specifically on T2DM patients providing the incidence for this patients as well as the evolution in their clinical characteristic and hospital outcomes and we identify the risk factors for IHM among this population. Nevertheless, beside those previously commented we should point out several limitations. Our data source was the SNHDD, an administrative database that relies on the information that physicians include in the discharge report and on manual coding on behalf of administrative staff. To our knowledge the ICD-9 codes for IE in the SNHDD have not been validated so far. However, results from three previous studies conducted in other countries, using ICD-9 codes in hospital discharge databases, suggested good accuracy for detection of endocarditis cases with reference to the revised Duke criteria [8, 28, 51]. The validity of the diabetes diagnosis in the SNHDD has been assessed in two previous studies, revealing a sensitivity of 55% and 63.7% and a specificity of approximately 97% [52, 53]. In a recent review of 12 studies (8 in US and 4 in Canada) using ICD-9 codes, specificity always showed high values (88% to 100%) with worse figures for sensitivity (26.9% to 100%) and the Kappa concordance index ranged from 0.6 to 0.9 [54]. We lack longitudinal information about diabetes and other variables. Another limitation of our investigation is that only up to around 70% had identified causative pathogens. As the SNHDD is anonymized we cannot confirm that a patient that suffered a previous IE hospitalization and who moved from one hospital to another could be counted twice. Also is possible that patients with IE in the second diagnosis position aren’t admitted for IE but for another disease and have history of IE. The number of patients that had IE as a second diagnosis was 3206 (19.3%) with similar values beside T2DM status (19.05% among T2DM and 19.34% among non-diabetic patients). In both groups over 90% of patients with a second diagnosis of IE the primary diagnosis were ICD-9 code 996.91 (Infection and inflammatory reaction due to cardiac device, implant, and graft” or valve disease ICD-9 codes (424.0, 424.1, 424.2, 424.3). Therefore we consider this error improbable and if exists of a very small magnitude that would not affect the main conclusions of our investigation as it affect similarly those with and without diabetes. HIV patients or those with drugs abuse suffering IE were excluded for two reasons, first because the clinical characteristics of IE among may be different to those without these conditions and secondly this is a very infrequent condition among T2DM patients (only 16/3436 [0.46%] vs. 415/13,190 [3.15%] among non-T2DM sufferers). Furthermore, we cannot rule out that coding practices may have changed somewhat over time. Finally, residual confounding not accounted for could be influencing the results of the multivariable analysis of the factors associated with in-hospital mortality.

## Conclusion

Incidence rates of IE in Spain, among those with and without T2DM, have increased during the period 2001–2015 with significantly higher incidence rates in the T2DM population. In both populations the age and comorbidity rose over time and the length of hospital stay decreased. Regarding IHM only a significant improvement was found among those with T2DM, remaining stable among those without this disease. In our population based study and after PSM we found that T2DM was not a predictor of IHM in IE. In the T2DM population predictors of higher IHM after IE included female older age, comorbidities, whereas Streptococci infection and COPD predicted lower IHM. However, given the limitations of hospital discharge databases prospective studies including detailed clinical data are necessary to confirm these conclusions.

## Supplementary information

**Additional file 1: Figure S1.** Flow chart of the patient selection.

**Additional file 2: Table S1.** Diagnosis and procedures analyzed with their corresponding ICD-9-CM codes.

**Additional file 3: Table S2.** Absolute standardized differences before and after Propensity Score Matching (PSM).

**Additional file 4: Table S3.** Baseline conditions by study periods among those patients suffering infective endocarditis with concomitant Type 2 Diabetes Mellitus.

**Additional file 5: Table S4.** Baseline conditions by study periods among those patients suffering infective endocarditis without concomitant Type 2 Diabetes Mellitus.

**Additional file 6: Table S5.** In hospital mortality by study variables of patients suffering infective endocarditis admitted to Spanish hospitals from 2001 to 2015 according to Type 2 Diabetes Mellitus status.

## Data Availability

According to the contract signed with the Spanish Ministry of Health and Social Services, which provided access to the databases from the Spanish National Hospital Database (*Conjunto Mínimo Basico de Datos*; CMBD), we cannot share the databases with any other investigator, and we have to destroy the databases once the investigation has concluded. Consequently, we cannot upload the databases to any public repository. However, any investigator can apply for access to the databases by filling out the questionnaire available at http://www.msssi.gob.es/estadEstudios/estadisticas/estadisticas/estMinisterio/SolicitudCMBDdocs/Formulario_Peticion_Datos_CMBD.pdf. All other relevant data are included in the paper.

## Abbreviations

CCI: Charlson Comorbidity Index
ICD-9-CM: International Classification of Diseases-Ninth Revision, Clinical Modification
DM: diabetes mellitus
IE: infective endocarditis
IHM: in-hospital mortality
LOHS: length of hospital stay
IRR, 95%CI: incidence rate ratio, 95% confidence interval
OR: odds ratio
SNHDD: Spanish National Hospital Discharge Data
PSM: propensity score matching
T2DM: type 2 diabetes mellitus

## References

[1] Murdoch DR, Corey GR, Hoen B, Miró JM, Fowler VG, Bayer AS (2009). Clinical presentation, etiology, and outcome of infective endocarditis in the 21st century: the International Collaboration on Endocarditis-Prospective Cohort Study. Arch Intern Med.

[2] Slipczuk L, Codolosa JN, Davila CD, Romero-Corral A, Yun J, Pressman GS (2013). Infective endocarditis epidemiology over five decades: a systematic review. PLoS ONE.

[3] Olmos C, Vilacosta I, Fernández-Pérez C, Bernal JL, Ferrera C, García-Arribas D (2017). The evolving nature of infective endocarditis in Spain: a population-based study (2003 to 2014). J Am Coll Cardiol.

[4] Bikdeli B, Wang Y, Kim N, Desai MM, Quagliarello V, Krumholz HM (2013). Trends in hospitalization rates and outcomes of endocarditis among Medicare beneficiaries. J Am Coll Cardiol.

[5] Soriguer F, Goday A, Bosch-Comas A, Bordiú E, Calle-Pascual A, Carmena R (2012). Prevalence of diabetes mellitus and impaired glucose regulation in Spain: the Di@bet.es Study. Diabetologia.

[6] NCD Risk Factor Collaboration (NCD-RisC) (1980). Worldwide trends in diabetes since 1980: a pooled analysis of 751 population-based studies with 4.4 million participants. Lancet.

[7] Basterra-Gortari FJ, Bes-Rastrollo M, Ruiz-Canela M, Gea A, Martinez-Gonzalez MÁ (2017). Prevalence of obesity and diabetes in Spanish adults 1987–2012. Med Clin.

[8] Toyoda N, Chikwe J, Itagaki S, Gelijns AC, Adams DH, Egorova NN (2017). Trends in infective endocarditis in California and New York State, 1998–2013. JAMA.

[9] Sy RW, Kritharides L (2010). Health care exposure and age in infective endocarditis: results of a contemporary population-based profile of 1536 patients in Australia. Eur Heart J.

[10] Delamaire M, Maugendre D, Moreno M, Le Goff MC, Allannic H, Genetet B (1997). Impaired leucocyte functions in diabetic patients. Diabet Med.

[11] Joshi N, Caputo GM, Weitekamp MR, Karchmer AW (1999). Infections in patients with diabetes mellitus. N Engl J Med.

[12] Chirillo F, Bacchion F, Pedrocco A, Scotton P, De Leo A, Rocco F (2010). Infective endocarditis in patients with diabetes mellitus. J Heart Valve Dis.

[13] Kourany WM, Miro JM, Moreno A, Corey GR, Pappas PA, Abrutyn E (2006). Influence of diabetes mellitus on the clinical manifestations and prognosis of infective endocarditis: a report from the International Collaboration on Endocarditis—Merged Database. Scand J Infect Dis.

[14] Tousoulis D, Kampoli AM, Stefanadis C (2012). Diabetes mellitus and vascular endothelial dysfunction: current perspectives. Curr Vasc Pharmacol.

[15] Eringa EC, Serne EH, Meijer RI, Schalkwijk CG, Houben AJHM, Stehouwer CDA (2013). Endothelial dysfunction in (pre)diabetes: characteristics, causative mechanisms and pathogenic role in type 2 diabetes. Rev Endocr Metab Disord.

[16] Strom BL, Abrutyn E, Berlin JA, Kinman JL, Feldman RS, Stolley PD (2000). Risk factors for infective endocarditis: oral hygiene and nondental exposures. Circulation.

[17] Østergaard L, Mogensen UM, Bundgaard JS, Dahl A, Wang A, Torp-Pedersen C (2019). Duration and complications of diabetes mellitus and the associated risk of infective endocarditis. Int J Cardiol.

[18] Critchley JA, Carey IM, Harris T, DeWilde S, Hosking FJ, Cook DG (2018). Glycemic control and risk of infections among people with type 1 or type 2 diabetes in a large primary care cohort study. Diabetes Care.

[19] Dayer MJ, Jones S, Prendergast B, Baddour LM, Lockhart PB, Thornhill MH (2015). Incidence of infective endocarditis in England, 2000–13: a secular trend, interrupted time-series analysis. Lancet.

[20] Movahed MR, Hashemzadeh M, Jamal MM (2007). Increased prevalence of infectious endocarditis in patients with type II diabetes mellitus. J Diabetes Complicat.

[21] Mistiaen WP (2018). What are the main predictors of in-hospital mortality in patients with infective endocarditis: a review. Scand Cardiovasc J.

[22] Wei XB, Liu YH, Huang JL, Chen XL, Yu DQ, Tan N (2018). Prediabetes and diabetes are both risk factors for adverse outcomes in infective endocarditis. Diabet Med.

[23] Olmos C, Vilacosta I, Pozo E, Fernández C, Sarriá C, López J (2014). Prognostic implications of diabetes in patients with left-sided endocarditis: findings from a large cohort study. Medicine.

[24] Lin CJ, Chua S, Chung SY, Hang CL, Tsai TH (2019). Diabetes mellitus: an independent risk factor of in-hospital mortality in patients with infective endocarditis in a New Era of clinical practice. Int J Environ Res Public Health.

[25] Chu VH, Cabell CH, Benjamin DK, Kuniholm EF, Fowler VG, Engemann J, Sexton DJ, Corey GR, Wang A (2004). Early predictors of in-hospital death in infective endocarditis. Circulation.

[26] Leone S, Ravasio V, Durante-Mangoni E, Crapis M, Carosi G, Scotton PG (2012). Epidemiology, characteristics, and outcome of infective endocarditis in Italy: the Italian Study on Endocarditis. Infection.

[27] Bishara J, Peled N, Samra Z, Sagie A, Leibovici L, Pitlik S (2004). Infective endocarditis in diabetic and non-diabetic patients. Scand J Infect Dis.

[28] Fedeli U, Schievano E, Buonfrate D, Pellizzer G, Spolaore P (2011). Increasing incidence and mortality of infective endocarditis: a population-based study through a record-linkage system. BMC Infect Dis.

[29] Duval X, Alla F, Doco-Lecompte T, Le Moing V, Delahaye F, Mainardi JL (2007). Diabetes mellitus and infective endocarditis: the insulin factor in patient morbidity and mortality. Eur Heart J.

[30] Nunes MCP, Guimarães-Júnior MH, Murta Pinto PHO, Coelho RMP, Souza Barros TL, Faleiro Maia NPA (2018). Outcomes of infective endocarditis in the current era: early predictors of a poor prognosis. Int J Infect Dis.

[31] Joffre J, Dumas G, Aegerter P, Dubée V, Bigé N, Preda G (2019). Epidemiology of infective endocarditis in French intensive care units over the 1997–2014 period-from CUB-Réa Network. Crit Care.

[32] Instituto Nacional de Gestión Sanitaria, Ministerio de Sanidad, Servicios Sociales e Igualdad. Conjunto Mínimo Básico de Datos, Hospitales del INSALUD. http://www.ingesa.mscbs.gob.es/estadEstudios/documPublica/pdf/CMBD-2001.pdf. Accessed 2 Jan 2019.

[33] Morita Y, Haruna T, Haruna Y, Nakane E, Yamaji Y, Hayashi H (2019). Thirty-day readmission after infective endocarditis: analysis from a nationwide readmission database. J Am Heart Assoc.

[34] Charlson ME, Pompei P, Ales KL, MacKenzie CR (1987). A new method of classifying prognostic comorbidity in longitudinal studies: development and validation. J Chronic Dis.

[35] Ministerio de Sanidad y Consumo. Análisis y desarrollo de los GDR en el Sistema Nacional de Salud. https://www.mscbs.gob.es/estadEstudios/estadisticas/docs/analisis.pdf. Accessed 2 Jan 2019.

[36] Austin PC (2011). An introduction to propensity score methods for reducing the effects of confounding in observational studies. Multivar Behav Res.

[37] D’Agostino RB (1998). Propensity score methods for bias reduction in the comparison of a treatment to a non-randomized control group. Stat Med.

[38] Leuven E, Sianesi B. PSMATCH2: Stata module to perform full Mahalanobis and propensity score matching, common support graphing, and covariate imbalance testing. https://econpapers.repec.org/software/bocbocode/s432001.htm. Accessed 9 Nov 2019.

[39] Austin PC (2011). Optimal caliper widths for propensity-score matching when estimating differences in means and differences in proportions in observational studies. Pharm Stat.

[40] Ministerio de Sanidad, Servicios Sociales e Igualdad. [Encuesta Nacional de Salud de España]. https://www.mscbs.gob.es/estadEstudios/estadisticas/encuestaNacional/aniosAnteriores.htm. Accessed 2 Jan 2019.

[41] Instituto Nacional de Estadística. Population estimates 2010. http://www.ine.es. Accessed 2 Jan 2019.

[42] Sullenberger AL, Avedissian LS, Kent SM (2005). Importance of transesophageal echocardiography in the evaluation of *Staphylococcus aureus* bacteremia. J Heart Valve Dis.

[43] Iversen K, Ihlemann N, Gill SU, Madsen T, Elming H, Jensen KT (2019). Partial oral versus intravenous antibiotic treatment of endocarditis. N Engl J Med.

[44] Bustamante-Munguira J, Mestres CA, Alvarez P, Figuerola-Tejerina A, Eiros Bachiller R, Gómez-Sánchez E (2018). Surgery for acute infective endocarditis: epidemiological data from a Spanish nationwide hospital-based registry. Interact Cardiovasc Thorac Surg.

[45] Duval X, Delahaye F, Alla F, Tattevin P, Obadia JF, Le Moing V (2012). Temporal trends in infective endocarditis in the context of prophylaxis guideline modifications: three successive population-based surveys. J Am Coll Cardiol.

[46] Ahtela E, Oksi J, Porela P, Ekström T, Rautava P, Kytö V (2019). Trends in occurrence and 30-day mortality of infective endocarditis in adults: population-based registry study in Finland. BMJ Open.

[47] Mata-Cases M, Franch-Nadal J, Real J, Mauricio D (2016). Glycaemic control and antidiabetic treatment trends in primary care centres in patients with type 2 diabetes mellitus during 2007–2013 in Catalonia: a population-based study. BMJ Open.

[48] Sambola A, Fernández-Hidalgo N, Almirante B, Roca I, González-Alujas T, Serra B (2010). Sex differences in native-valve infective endocarditis in a single tertiary-care hospital. Am J Cardiol.

[49] Aksoy O, Meyer LT, Cabell CH, Kourany WM, Pappas PA, Sexton DJ (2007). Gender differences in infective endocarditis: pre- and co-morbid conditions lead to different management and outcomes in female patients. Scand J Infect Dis.

[50] Liang F, Song B, Liu R, Yang L, Tang H, Li Y (2016). Optimal timing for early surgery in infective endocarditis: a meta-analysis. Interact Cardiovasc Thorac Surg.

[51] Schneeweiss S, Robicsek A, Scranton R, Zuckerman D, Solomon DH (2007). Veteran’s affairs hospital discharge databases coded serious bacterial infections accurately. J Clin Epidemiol.

[52] Ribera A, Marsal JR, Ferreira-González I, Cascant P, Cascant P, Pons JM, Mitjavila F (2008). Predicting in-hospital mortality with coronary bypass surgery using hospital discharge data: comparison with a prospective observational study. Rev Esp Cardiol.

[53] Rodrigo-Rincón I, Martin-Vizcaíno MP, Tirapu-León B, Zabalza-López P, Abad-Vicente FJ, Merino-Peralta A (2016). Usefulness of administrative databases for risk adjustment of adverse events in surgical patients. Cir Esp.

[54] Khokhar B, Jette N, Metcalfe A, Cunningham CT, Quan H, Kaplan GG (2016). Systematic review of validated case definitions for diabetes in ICD-9-coded and ICD-10-coded data in adult populations. BMJ Open.

