# Supplementary material for: Infective endocarditis according to type 2 diabetes mellitus status: an observational study in Spain, 2001–2015
Source: Cardiovasc Diabetol. 2019 Nov 21;18:161. doi: 10.1186/s12933-019-0968-0 (PMC6868776; doi:10.1186/s12933-019-0968-0)
Supplement: Supplementary file 1 — Additional file 1: Figure S1. Flow chart of the patient selection. [file 12933_2019_968_MOESM1_ESM.docx]

Figure S1. Flow chart of the patient selection

Not co-existing with HIV or drugs abuse

N=16.626 (97.5%)

STUDY SAMPLE

No ICD-9 codes for T2DM in any diagnosis positions (1 to 14).

N=13,190 (89.3%)

ICD-9 codes for T2DM in any diagnosis positions (1 to 14).

N=3,436 (20.7%)

EXCLUDED

EXCLUDED

INCLUDED

EXCLUDED

EXCLUDED

INCLUDED

INCLUDED

INCLUDED

Co-existing with HIV or drugs abuse

N=431 (2.5%)

Age under 40 years.

N=2,676(12.8%)

Index IE

N=17.057 (93.5%)

Age 40 years or over.

N=18,239 (87.2%)

Cases with a previous admission with IE.

N=1,182 (6.5%)

SNHDD from 2001 to 2015 with a ICD-9 codes for IE in any diagnosis positions (1 to 14).

N=33,745

SNHDD from 2001 to 2015 with a code for IE in first and second diagnosis positions.

N=20,915

SNHDD from 2001 to 2015 with a code for IE in diagnosis positions 3 to 14.

N=12,830
