# Supplementary material for: Infective endocarditis according to type 2 diabetes mellitus status: an observational study in Spain, 2001–2015
Source: Cardiovasc Diabetol. 2019 Nov 21;18:161. doi: 10.1186/s12933-019-0968-0 (PMC6868776; doi:10.1186/s12933-019-0968-0)
Supplement: Supplementary file 2 — Additional file 2: Table S1. Diagnosis and procedures analyzed with their corresponding ICD-9-CM codes. [file 12933_2019_968_MOESM2_ESM.docx]

Table S1. Diagnosis and procedures analyzed with their corresponding ICD-9-CM codes.

|  | **ICD-9-CM codes** |
| --- | --- |
| Prosthetic valve carriers | V43.3 |
| Previous mitral valve disease | 394.x, 396.x, 424.0 |
| Previous aortic valve disease | 395.x, 396.x, 424.1 |
| Congestive heart failure | 398.91,402.01,402.11,402.91,404.01, 404.03,404.11,404.13,404.91,404.93,425.4–425.9, 428.x |
| Septic arterial embolism | 449 |
| Dementia | 290.x, 294.x |
| Acute renal disease | 584, 584.5-584.9 |
| Chronic renal disease | 585 |
| Ischemic heart disease | 410.x-414.x |
| Chronic obstructive pulmonary disease | 490, 491, 491.0, 491.1, 491.2x, 491.8, 491.9, 492, 492.0, 492.8, 496 |
| Atrial fibrillation | 427.31 |
| Shock | 785.5x |
| Periannular complications/atrioventricular block | 429.5, 429.6, 426.0, 426.12, 426.13 |
| Heart valve surgery | 35.21, 35.22, 35.23, 35.24 |
| Dialysis | 39.95 |
| Pacemaker implantation | 37.70-37.74; 37.80-37.83 |
| Mechanical ventilation | 93.90, 96.7, 96.70, 96.71, 96.72 |
| Coagulase-negative staphylococci | 041.19 |
| *Staphylococcus aureus* | 482.42 |
| Streptococci | 482.30 |
| Enterococci | 041.04 |
| *Streptococcus pneumonia* | 481 |
| Anaerobes | 482.21 |
| Gram-negative bacilli | 482.83 |
| Candidiasis/Aspergillosis | 112.4, 117.3 |
