# Supplementary material for: Infective endocarditis according to type 2 diabetes mellitus status: an observational study in Spain, 2001–2015
Source: Cardiovasc Diabetol. 2019 Nov 21;18:161. doi: 10.1186/s12933-019-0968-0 (PMC6868776; doi:10.1186/s12933-019-0968-0)
Supplement: Supplementary file 3 — Additional file 3: Table S2. Absolute standardized differences before and after Propensity Score Matching (PSM). [file 12933_2019_968_MOESM3_ESM.docx]

Table S2. Absolute standardized differences before and after Propensity Score Matching (PSM).

|  | | Before matching (PSM) | | |  | After PSM matching | | |  |
| --- | --- | --- | --- | --- | --- | --- | --- | --- | --- |
|  | | T2DM | Non T2DM | ASD | P value | T2DM | Non T2DM | ASD | P value |
| Time period. n (%) | 2001-2003 | 345(10.0) | 2.019(15.3) | 15.9 | <0.001 | 345(10.0) | 371(10.8) | 2.5 | 0.277 |
|  | 2004-2006 | 486(14.1) | 2.128(16.1) | 5.6 |  | 486(14.1) | 456(13.3) | 2.5 |  |
|  | 2007-2009 | 603(17.6) | 2.304(17.5) | 0.2 |  | 603(17.6) | 576(16.8) | 2.1 |  |
|  | 2010-2012 | 962(28.0) | 3.165(24.0) | 9.1 |  | 962(28.0) | 928(27.0) | 2.2 |  |
|  | 2013-2015 | 1.040(30.3) | 3.574(27.1) | 7 |  | 1.040(30.3) | 1.105(32.2) | 4.1 |  |
| Sex. n (%) | Male | 2.196(63.9) | 8.865(67.2) | 6.9 | <0.001 | 2.196(63.9) | 2.175(63.3) | 1.3 | 0.599 |
|  | Female | 1.240(36.1) | 4.325(32.8) | 6.9 |  | 1.240(36.1) | 1.261(36.7) | 1.3 |  |
| Age. mean (SD) | | 70.8(10.2) | 67.9(12.4) | 25.63 | <0.001 | 70.8(10.2) | 70.3(11.8) | 4.65 | 0.154 |
| Age groups. n (%) | 40-66 years old | 1.030(30.0) | 5.437(41.2) | 23.6 | <0.001 | 1.030(30.0) | 1.067(31.1) | 2.3 | 0.001 |
|  | 67-75 years old | 1.153(33.6) | 3.547(26.9) | 14.6 |  | 1.153(33.6) | 1.014(29.5) | 8.7 |  |
|  | ≥76 years old | 1.253(36.5) | 4.206(31.9) | 9.7 |  | 1.253(36.5) | 1.355(39.4) | 6.1 |  |
| CCI. mean (SD) | | 1.1(0.8) | 0.9(0.8) | 20.44 | <0.001 | 1.1(0.8) | 1.1(0.8) | 1.02 | 0.673 |
| Prosthetic valve carriers. n (%) | | 333(9.7) | 1.220(9.3) | 1.5 | 0.428 | 333(9.7) | 306(8.9) | 2.7 | 0.262 |
| Previous mitral valve disease. n (%) | | 912(26.5) | 3.971(30.1) | 7.9 | <0.001 | 912(26.5) | 908(26.4) | 0.3 | 0.913 |
| Previous aortic valve disease. n (%) | | 869(25.3) | 3.880(29.4) | 9.3 | <0.001 | 869(25.3) | 846(24.6) | 1.5 | 0.521 |
| Congestive heart failure. n (%) | | 972(28.3) | 3.666(27.8) | 1.1 | 0.565 | 972(28.3) | 935(27.2) | 2.4 | 0.319 |
| Septic arterial embolism. n (%) | | 39(1.1) | 164(1.2) | 1 | 0.607 | 39(1.1) | 46(1.3) | 1.8 | 0.445 |
| Dementia. n (%) | | 61(1.8) | 153(1.2) | 5.1 | 0.004 | 61(1.8) | 56(1.6) | 1.1 | 0.641 |
| Acute renal disease. n (%) | | 617(18.0) | 2.432(18.4) | 1.2 | 0.516 | 617(18.0) | 598(17.4) | 1.4 | 0.548 |
| Chronic renal disease. n (%) | | 592(17.2) | 1.329(10.1) | 20.9 | <0.001 | 592(17.2) | 536(15.6) | 4.4 | 0.068 |
| Ischemic heart disease. n (%) | | 627(18.3) | 1.467(11.1) | 20.2 | <0.001 | 627(18.3) | 611(17.8) | 1.2 | 0.616 |
| COPD. n (%) | | 602(17.5) | 2.167(16.4) | 2.9 | 0.126 | 602(17.5) | 643(18.7) | 3.1 | 0.199 |
| Atrial fibrillation. n (%) | | 863(25.1) | 2.916(22.1) | 7.1 | <0.001 | 863(25.1) | 867(25.2) | 0.3 | 0.911 |
| Shock. n (%) | | 239(7.0) | 1.099(8.3) | 5.2 | 0.008 | 239(7.0) | 221(6.4) | 2.1 | 0.385 |
| Periannular complications / atrioventricular block. n (%) | | 129(3.8) | 600(4.6) | 4 | 0.043 | 129(3.8) | 165(4.8) | 5.2 | 0.032 |
| Heart valve surgery. n (%) | | 479(13.9) | 2.630(19.9) | 16 | <0.001 | 479(13.9) | 595(17.3) | 9.3 | <0.001 |
| Dialysis. n (%) | | 248(7.2) | 761(5.8) | 5.9 | <0.001 | 248(7.2) | 203(5.9) | 5.3 | 0.028 |
| Pacemaker implantation. n (%) | | 89(2.6) | 356(2.7) | 0.7 | 0.725 | 89(2.6) | 86(2.5) | 0.6 | 0.818 |
| Mechanical ventilation. n (%) | | 410(11.9) | 1.774(13.5) | 4.6 | 0.019 | 410(11.9) | 412(12.0) | 0.2 | 0.941 |
| Coagulase-negative staphylococci. n (%) | | 441(12.8) | 1.463(11.1) | 5.4 | 0.004 | 441(12.8) | 399(11.6) | 3.7 | 0.122 |
| Staphylococcus aureus. n (%) | | 504(14.7) | 1.685(12.8) | 5.5 | 0.003 | 504(14.7) | 452(13.2) | 4.4 | 0.070 |
| Streptococci. n (%) | | 649(18.9) | 2.897(22.0) | 7.6 | <0.001 | 649(18.9) | 749(21.8) | 7.2 | 0.003 |
| Enterococci. n (%) | | 557(16.2) | 1.887(14.3) | 5.3 | 0.005 | 557(16.2) | 488(14.2) | 5.6 | 0.020 |
| Streptococcus pneumoniae. n (%) | | 15(0.4) | 58(0.4) | 0 | 0.980 | 15(0.4) | 13(0.4) | 0.9 | 0.705 |
| Anaerobes. n (%) | | 21(0.6) | 71(0.5) | 1 | 0.608 | 21(0.6) | 19(0.6) | 0.8 | 0.751 |
| Gram-negative bacilli. n (%) | | 264(7.7) | 1.012(7.7) | 0 | 0.983 | 264(7.7) | 260(7.6) | 0.4 | 0.856 |
| Candidiasis / Aspergillosis. n (%) | | 3(0.1) | 32(0.2) | 3.8 | 0.077 | 3(0.1) | 11(0.3) | 5.2 | 0.032 |
| Readmissions. n (%) | | 687(20.0) | 2.091(15.9) | 10.8 | <0.001 | 687(20.0) | 632(18.4) | 4.1 | 0.092 |
| Length of hospital stay. mean (SD) | | 27.17(20.4) | 27.51(22.1) | 3.4 | 0.405 | 27.17(20.4) | 27.43(22.1) | 2.3 | 0.616 |
| In-hospital mortality. n (%) | | 714(20.8) | 2.562(19.4) | 1.63 | 0.075 | 714(20.8) | 682(19.9) | 1.21 | 0.337 |
| Cost. mean (SD) | | 13.230.4 | 14.452.0 | 10.61 | <0.001 | 13.230.4 | 14.045.3 | 7.12 | 0.003 |
|  |  | (10.000.7) | (12.845.6) |  |  | (10.000.7) | (12.713.2) |  |  |

ASD Absolute Standard Difference
