# Supplementary material for: Infective endocarditis according to type 2 diabetes mellitus status: an observational study in Spain, 2001–2015
Source: Cardiovasc Diabetol. 2019 Nov 21;18:161. doi: 10.1186/s12933-019-0968-0 (PMC6868776; doi:10.1186/s12933-019-0968-0)
Supplement: Supplementary file 4 — Additional file 4: Table S3. Baseline conditions by study periods among those patients suffering infective endocarditis with concomitant Type 2 Diabetes Mellitus. [file 12933_2019_968_MOESM4_ESM.docx]

Table S3. Baseline conditions by study periods among those patients suffering infective endocarditis with concomitant Type 2 Diabetes Mellitus.

|  | |  | | | | | |
| --- | --- | --- | --- | --- | --- | --- | --- |
|  |  | **2001-2003** | **2004-2006** | **2007-2009** | **2010-2012** | **2013-2015** | **P-value** |
| Sex, n (%) | Male | 215(62,32) | 293(60,29) | 370(61,36) | 614(63,83) | 704(67,69) | 0,023 |
|  | Female | 130(37,68) | 193(39,71) | 233(38,64) | 348(36,17) | 336(32,31) |  |
| Age groups, n (%) | 40-66 years old | 129(37,39) | 170(34,98) | 189(31,34) | 255(26,51) | 287(27,6) | 0,000 |
|  | 67-75 years old | 132(38,26) | 178(36,63) | 203(33,67) | 302(31,39) | 338(32,5) |  |
|  | ≥76 years old | 84(24,35) | 138(28,4) | 211(34,99) | 405(42,1) | 415(39,9) |  |
| Prosthetic valve carriers, n (%) | No | 318(92,17) | 445(91,56) | 546(90,55) | 856(88,98) | 938(90,19) | 0,375 |
|  | Yes | 27(7,83) | 41(8,44) | 57(9,45) | 106(11,02) | 102(9,81) |  |
| Previous mitral valve disease, n (%) | No | 246(71,3) | 364(74,9) | 447(74,13) | 691(71,83) | 776(74,62) | 0,478 |
|  | Yes | 99(28,7) | 122(25,1) | 156(25,87) | 271(28,17) | 264(25,38) |  |
| Previous aortic valve disease, n (%) | No | 270(78,26) | 372(76,54) | 454(75,29) | 711(73,91) | 760(73,08) | 0,280 |
|  | Yes | 75(21,74) | 114(23,46) | 149(24,71) | 251(26,09) | 280(26,92) |  |
| Congestive heart failure, n (%) | No | 268(77,68) | 357(73,46) | 441(73,13) | 674(70,06) | 724(69,62) | 0,027 |
|  | Yes | 77(22,32) | 129(26,54) | 162(26,87) | 288(29,94) | 316(30,38) |  |
| Septic arterial embolism, n (%) | No | 345(100) | 486(100) | 603(100) | 942(97,92) | 1021(98,17) | 0,000 |
|  | Yes | 0(0) | 0(0) | 0(0) | 20(2,08) | 19(1,83) |  |
| Dementia, n (%) | No | 336(97,39) | 479(98,56) | 594(98,51) | 941(97,82) | 1025(98,56) | 0,472 |
|  | Yes | 9(2,61) | 7(1,44) | 9(1,49) | 21(2,18) | 15(1,44) |  |
| Acute renal disease, n (%) | No | 296(85,8) | 408(83,95) | 515(85,41) | 776(80,67) | 824(79,23) | 0,003 |
|  | Yes | 49(14,2) | 78(16,05) | 88(14,59) | 186(19,33) | 216(20,77) |  |
| Chronic renal disease, n (%) | No | 316(91,59) | 441(90,74) | 518(85,9) | 759(78,9) | 810(77,88) | 0,000 |
|  | Yes | 29(8,41) | 45(9,26) | 85(14,1) | 203(21,1) | 230(22,12) |  |
| Ischemic heart disease, n (%) | No | 291(84,35) | 402(82,72) | 499(82,75) | 791(82,22) | 826(79,42) | 0,185 |
|  | Yes | 54(15,65) | 84(17,28) | 104(17,25) | 171(17,78) | 214(20,58) |  |
| COPD, n (%) | No | 294(85,22) | 407(83,74) | 503(83,42) | 793(82,43) | 837(80,48) | 0,234 |
|  | Yes | 51(14,78) | 79(16,26) | 100(16,58) | 169(17,57) | 203(19,52) |  |
| Atrial fibrillation, n (%) | No | 258(74,78) | 372(76,54) | 462(76,62) | 724(75,26) | 757(72,79) | 0,383 |
|  | Yes | 87(25,22) | 114(23,46) | 141(23,38) | 238(24,74) | 283(27,21) |  |
| Shock, n (%) | No | 318(92,17) | 445(91,56) | 557(92,37) | 898(93,35) | 979(94,13) | 0,340 |
|  | Yes | 27(7,83) | 41(8,44) | 46(7,63) | 64(6,65) | 61(5,87) |  |
| Periannular complications / atrioventricular block, n (%) | No | 339(98,26) | 470(96,71) | 588(97,51) | 920(95,63) | 990(95,19) | 0,026 |
|  | Yes | 6(1,74) | 16(3,29) | 15(2,49) | 42(4,37) | 50(4,81) |  |
| Heart valve surgery, n (%) | No | 313(90,72) | 413(84,98) | 515(85,41) | 840(87,32) | 876(84,23) | 0,025 |
|  | Yes | 32(9,28) | 73(15,02) | 88(14,59) | 122(12,68) | 164(15,77) |  |
| Dialysis, n (%) | No | 322(93,33) | 451(92,8) | 558(92,54) | 894(92,93) | 963(92,6) | 0,990 |
|  | Yes | 23(6,67) | 35(7,2) | 45(7,46) | 68(7,07) | 77(7,4) |  |
| Pacemaker implantation, n (%) | No | 344(99,71) | 477(98,15) | 592(98,18) | 923(95,95) | 1011(97,21) | 0,001 |
|  | Yes | 1(0,29) | 9(1,85) | 11(1,82) | 39(4,05) | 29(2,79) |  |
| Mechanical ventilation, n (%) | No | 304(88,12) | 438(90,12) | 532(88,23) | 854(88,77) | 898(86,35) | 0,252 |
|  | Yes | 41(11,88) | 48(9,88) | 71(11,77) | 108(11,23) | 142(13,65) |  |
| Coagulase-negative staphylococci, n (%) | No | 317(91,88) | 425(87,45) | 550(91,21) | 814(84,62) | 889(85,48) | 0,000 |
|  | Yes | 28(8,12) | 61(12,55) | 53(8,79) | 148(15,38) | 151(14,52) |  |
| *Staphylococcus aureus,* n (%) | No | 285(82,61) | 416(85,6) | 515(85,41) | 832(86,49) | 884(85) | 0,527 |
|  | Yes | 60(17,39) | 70(14,4) | 88(14,59) | 130(13,51) | 156(15) |  |
| Streptococci, n (%) | No | 266(77,1) | 379(77,98) | 482(79,93) | 796(82,74) | 864(83,08) | 0,021 |
|  | Yes | 79(22,9) | 107(22,02) | 121(20,07) | 166(17,26) | 176(16,92) |  |
| Enterococci, n (%) | No | 301(87,25) | 425(87,45) | 494(81,92) | 803(83,47) | 856(82,31) | 0,025 |
|  | Yes | 44(12,75) | 61(12,55) | 109(18,08) | 159(16,53) | 184(17,69) |  |
| *Streptococcus pneumoniae,* n (%) | No | 344(99,71) | 482(99,18) | 601(99,67) | 959(99,69) | 1035(99,52) | 0,665 |
|  | Yes | 1(0,29) | 4(0,82) | 2(0,33) | 3(0,31) | 5(0,48) |  |
| Anaerobes, n (%) | No | 344(99,71) | 485(99,79) | 600(99,5) | 956(99,38) | 1030(99,04) | 0,388 |
|  | Yes | 1(0,29) | 1(0,21) | 3(0,5) | 6(0,62) | 10(0,96) |  |
| Gram-negative bacilli, n (%) | No | 332(96,23) | 450(92,59) | 557(92,37) | 883(91,79) | 950(91,35) | 0,055 |
|  | Yes | 13(3,77) | 36(7,41) | 46(7,63) | 79(8,21) | 90(8,65) |  |
| Candidiasis / Aspergillosis, n (%) | No | 345(100) | 485(99,79) | 602(99,83) | 962(100) | 1039(99,9) | 0,670 |
|  | Yes | 0(0) | 1(0,21) | 1(0,17) | 0(0) | 1(0,1) |  |
| Readmissions, n (%) | No | 287(83,19) | 395(81,28) | 487(80,76) | 763(79,31) | 817(78,56) | 0,338 |
|  | Yes | 58(16,81) | 91(18,72) | 116(19,24) | 199(20,69) | 223(21,44) |  |
| In-hospital mortality, n (%) | No | 258(74,78) | 385(79,22) | 468(77,61) | 753(78,27) | 858(82,5) | 0,015 |
|  | Yes | 87(25,22) | 101(20,78) | 135(22,39) | 209(21,73) | 182(17,5) |  |
