# Supplementary material for: Infective endocarditis according to type 2 diabetes mellitus status: an observational study in Spain, 2001–2015
Source: Cardiovasc Diabetol. 2019 Nov 21;18:161. doi: 10.1186/s12933-019-0968-0 (PMC6868776; doi:10.1186/s12933-019-0968-0)
Supplement: Supplementary file 5 — Additional file 5: Table S4. Baseline conditions by study periods among those patients suffering infective endocarditis without concomitant Type 2 Diabetes Mellitus. [file 12933_2019_968_MOESM5_ESM.docx]

Table S4. Baseline conditions by study periods among those patients suffering infective endocarditis without concomitant Type 2 Diabetes Mellitus.

|  | |  | | | | | |
| --- | --- | --- | --- | --- | --- | --- | --- |
|  |  | 2001-2003 | 2004-2006 | 2007-2009 | 2010-2012 | 2013-2015 | p-value |
| Sex, n (%) | Male | 1352(66,96) | 1424(66,92) | 1518(65,89) | 2128(67,24) | 2443(68,35) | 0,393 |
|  | Female | 667(33,04) | 704(33,08) | 786(34,11) | 1037(32,76) | 1131(31,65) |  |
| Age groups, n (%) | 40-66 years old | 895(44,33) | 995(46,76) | 992(43,06) | 1229(38,83) | 1326(37,1) | 0,000 |
|  | 67-75 years old | 636(31,5) | 619(29,09) | 611(26,52) | 788(24,9) | 893(24,99) |  |
|  | ≥76 years old | 488(24,17) | 514(24,15) | 701(30,43) | 1148(36,27) | 1355(37,91) |  |
| Prosthetic valve carriers, n (%) | No | 1849(91,58) | 1937(91,02) | 2099(91,1) | 2871(90,71) | 3214(89,93) | 0,280 |
|  | Yes | 170(8,42) | 191(8,98) | 205(8,9) | 294(9,29) | 360(10,07) |  |
| Previous mitral valve disease, n (%) | No | 1479(73,25) | 1512(71,05) | 1623(70,44) | 2131(67,33) | 2474(69,22) | 0,000 |
|  | Yes | 540(26,75) | 616(28,95) | 681(29,56) | 1034(32,67) | 1100(30,78) |  |
| Previous aortic valve disease, n (%) | No | 1508(74,69) | 1526(71,71) | 1631(70,79) | 2193(69,29) | 2452(68,61) | 0,000 |
|  | Yes | 511(25,31) | 602(28,29) | 673(29,21) | 972(30,71) | 1122(31,39) |  |
| Congestive heart failure, n (%) | No | 1537(76,13) | 1582(74,34) | 1721(74,7) | 2226(70,33) | 2458(68,77) | 0,000 |
|  | Yes | 482(23,87) | 546(25,66) | 583(25,3) | 939(29,67) | 1116(31,23) |  |
| Septic arterial embolism, n (%) | No | 2019(100) | 2128(100) | 2304(100) | 3095(97,79) | 3480(97,37) | 0,000 |
|  | Yes | 0(0) | 0(0) | 0(0) | 70(2,21) | 94(2,63) |  |
| Dementia, n (%) | No | 1995(98,81) | 2103(98,83) | 2281(99) | 3124(98,7) | 3534(98,88) | 0,894 |
|  | Yes | 24(1,19) | 25(1,17) | 23(1) | 41(1,3) | 40(1,12) |  |
| Acute renal disease, n (%) | No | 1792(88,76) | 1812(85,15) | 1923(83,46) | 2520(79,62) | 2711(75,85) | 0,000 |
|  | Yes | 227(11,24) | 316(14,85) | 381(16,54) | 645(20,38) | 863(24,15) |  |
| Chronic renal disease, n (%) | No | 1922(95,2) | 2011(94,5) | 2100(91,15) | 2738(86,51) | 3090(86,46) | 0,000 |
|  | Yes | 97(4,8) | 117(5,5) | 204(8,85) | 427(13,49) | 484(13,54) |  |
| Ischemic heart disease, n (%) | No | 1842(91,23) | 1955(91,87) | 2057(89,28) | 2753(86,98) | 3116(87,19) | 0,000 |
|  | Yes | 177(8,77) | 173(8,13) | 247(10,72) | 412(13,02) | 458(12,81) |  |
| COPD, n (%) | No | 1770(87,67) | 1792(84,21) | 1949(84,59) | 2582(81,58) | 2930(81,98) | 0,000 |
|  | Yes | 249(12,33) | 336(15,79) | 355(15,41) | 583(18,42) | 644(18,02) |  |
| Atrial fibrillation, n (%) | No | 1634(80,93) | 1679(78,9) | 1849(80,25) | 2452(77,47) | 2660(74,43) | 0,000 |
|  | Yes | 385(19,07) | 449(21,1) | 455(19,75) | 713(22,53) | 914(25,57) |  |
| Shock, n (%) | No | 1867(92,47) | 1963(92,25) | 2104(91,32) | 2870(90,68) | 3287(91,97) | 0,113 |
|  | Yes | 152(7,53) | 165(7,75) | 200(8,68) | 295(9,32) | 287(8,03) |  |
| Periannular complications / atrioventricular block, n (%) | No | 1962(97,18) | 2050(96,33) | 2201(95,53) | 3001(94,82) | 3376(94,46) | 0,000 |
|  | Yes | 57(2,82) | 78(3,67) | 103(4,47) | 164(5,18) | 198(5,54) |  |
| Heart valve surgery, n (%) | No | 1657(82,07) | 1688(79,32) | 1856(80,56) | 2547(80,47) | 2812(78,68) | 0,029 |
|  | Yes | 362(17,93) | 440(20,68) | 448(19,44) | 618(19,53) | 762(21,32) |  |
| Dialysis, n (%) | No | 1940(96,09) | 2016(94,74) | 2169(94,14) | 2959(93,49) | 3345(93,59) | 0,001 |
|  | Yes | 79(3,91) | 112(5,26) | 135(5,86) | 206(6,51) | 229(6,41) |  |
| Pacemaker implantation, n (%) | No | 1984(98,27) | 2075(97,51) | 2243(97,35) | 3065(96,84) | 3467(97,01) | 0,024 |
|  | Yes | 35(1,73) | 53(2,49) | 61(2,65) | 100(3,16) | 107(2,99) |  |
| Mechanical ventilation, n (%) | No | 1790(88,66) | 1857(87,27) | 1986(86,2) | 2702(85,37) | 3081(86,21) | 0,011 |
|  | Yes | 229(11,34) | 271(12,73) | 318(13,8) | 463(14,63) | 493(13,79) |  |
| Coagulase-negative staphylococci, n (%) | No | 1852(91,73) | 1931(90,74) | 2060(89,41) | 2750(86,89) | 3134(87,69) | 0,000 |
|  | Yes | 167(8,27) | 197(9,26) | 244(10,59) | 415(13,11) | 440(12,31) |  |
| *Staphylococcus aureus,* n (%) | No | 1774(87,87) | 1851(86,98) | 2025(87,89) | 2738(86,51) | 3117(87,21) | 0,520 |
|  | Yes | 245(12,13) | 277(13,02) | 279(12,11) | 427(13,49) | 457(12,79) |  |
| Streptococci, n (%) | No | 1588(78,65) | 1625(76,36) | 1787(77,56) | 2480(78,36) | 2813(78,71) | 0,253 |
|  | Yes | 431(21,35) | 503(23,64) | 517(22,44) | 685(21,64) | 761(21,29) |  |
| Enterococci, n (%) | No | 1822(90,24) | 1874(88,06) | 1966(85,33) | 2682(84,74) | 2959(82,79) | 0,000 |
|  | Yes | 197(9,76) | 254(11,94) | 338(14,67) | 483(15,26) | 615(17,21) |  |
| *Streptococcus pneumoniae,* n (%) | No | 2006(99,36) | 2119(99,58) | 2295(99,61) | 3152(99,59) | 3560(99,61) | 0,679 |
|  | Yes | 13(0,64) | 9(0,42) | 9(0,39) | 13(0,41) | 14(0,39) |  |
| Anaerobes, n (%) | No | 2016(99,85) | 2118(99,53) | 2296(99,65) | 3147(99,43) | 3542(99,1) | 0,003 |
|  | Yes | 3(0,15) | 10(0,47) | 8(0,35) | 18(0,57) | 32(0,9) |  |
| Gram-negative bacilli, n (%) | No | 1924(95,29) | 1985(93,28) | 2145(93,1) | 2897(91,53) | 3227(90,29) | 0,000 |
|  | Yes | 95(4,71) | 143(6,72) | 159(6,9) | 268(8,47) | 347(9,71) |  |
| Candidiasis / Aspergillosis, n (%) | No | 2017(99,9) | 2121(99,67) | 2298(99,74) | 3157(99,75) | 3565(99,75) | 0,657 |
|  | Yes | 2(0,1) | 7(0,33) | 6(0,26) | 8(0,25) | 9(0,25) |  |
| Readmissions, n (%) | No | 1719(85,14) | 1814(85,24) | 1950(84,64) | 2639(83,38) | 2977(83,3) | 0,128 |
|  | Yes | 300(14,86) | 314(14,76) | 354(15,36) | 526(16,62) | 597(16,7) |  |
| In-hospital mortality, n (%) | No | 1588(78,65) | 1731(81,34) | 1865(80,95) | 2527(79,84) | 2917(81,62) | 0,053 |
|  | Yes | 431(21,35) | 397(18,66) | 439(19,05) | 638(20,16) | 657(18,38) |  |
