# Supplementary material for: Infective endocarditis according to type 2 diabetes mellitus status: an observational study in Spain, 2001–2015
Source: Cardiovasc Diabetol. 2019 Nov 21;18:161. doi: 10.1186/s12933-019-0968-0 (PMC6868776; doi:10.1186/s12933-019-0968-0)
Supplement: Supplementary file 6 — Additional file 6: Table S5. In hospital mortality by study variables of patients suffering infective endocarditis admitted to Spanish hospitals from 2001 to 2015 according to Type 2 Diabetes Mellitus status. [file 12933_2019_968_MOESM6_ESM.docx]

Table S5. In hospital mortality by study variables of patients suffering infective endocarditis admitted to Spanish hospitals from 2001 to 2015 according to Type 2 Diabetes Mellitus status.

| VARIABLE | CATHEGORIES | T2DM | P value | Non T2DM | P value |
| --- | --- | --- | --- | --- | --- |
| Sex, n (%) | Male | 414(18.9) | <0.001 | 405(18.6) | 0.018 |
|  | Female | 300(24.2) |  | 277(22.0) |  |
| Age groups, n (%) | 40-66 years old | 150(14.6) | <0.001 | 154(14.4) | <0.001 |
|  | 67-75 years old | 228(19.8) |  | 194(19.1) |  |
|  | ≥76 years old | 336(26.8) |  | 334(24.7) |  |
| Prosthetic valve carriers, n (%) | Yes | 57(17.1) | 0.083 | 56(18.3) | 0.477 |
|  | No | 657(21.17) |  | 626(20.00) |  |
| Previous mitral valve disease, n (%) | Yes | 190(20.8) | 0.963 | 180(19.8) | 0.983 |
|  | No | 524(20.76) |  | 502(19.86) |  |
| Previous aortic valve disease, n (%) | Yes | 184(21.2) | 0.741 | 161(19.0) | 0.492 |
|  | No | 530(20.65) |  | 521(20.12) |  |
| Congestive heart failure, n (%) | Yes | 274(28.2) | <0.001 | 284(30.4) | <0.001 |
|  | No | 440(17.86) |  | 398(15.91) |  |
| Septic arterial embolism, n (%) | Yes | 9(23.1) | 0.722 | 10(21.7) | 0.746 |
|  | No | 705(20.75) |  | 672(19.82) |  |
| Dementia, n (%) | Yes | 18(29.5) | 0.090 | 16(28.6) | 0.099 |
|  | No | 696(20.62) |  | 666(19.7) |  |
| Acute renal disease, n (%) | Yes | 241(39.1) | <0.001 | 224(37.5) | <0.001 |
|  | No | 473(16.78) |  | 458(16.14) |  |
| Chronic renal disease, n (%) | Yes | 161(27.2) | <0.001 | 135(25.2) | 0.001 |
|  | No | 553(19.44) |  | 547(18.86) |  |
| Ischemic heart disease, n (%) | Yes | 167(26.6) | <0.001 | 143(23.4) | 0.015 |
|  | No | 547(19.47) |  | 539(19.08) |  |
| COPD, n (%) | Yes | 129(21.4) | 0.666 | 138(21.5) | 0.255 |
|  | No | 585(20.64) |  | 544(19.48) |  |
| Atrial fibrillation, n (%) | Yes | 176(20.4) | 0.747 | 201(23.2) | 0.004 |
|  | No | 538(20.91) |  | 481(18.72) |  |
| Shock, n (%) | Yes | 169(70.7) | <0.001 | 157(71.0) | <0.001 |
|  | No | 545(17.05) |  | 525(16.33) |  |
| Periannular complications / atrioventricular block, n (%) | Yes | 31(24.0) | 0.354 | 38(23.0) | 0.294 |
|  | No | 683(20.65) |  | 644(19.69) |  |
| Heart valve surgery, n (%) | Yes | 108(22.6) | 0.304 | 149(25.0) | <0.001 |
|  | No | 606(20.49) |  | 533(18.76) |  |
| Dialysis, n (%) | Yes | 115(46.4) | <0.001 | 94(46.3) | <0.001 |
|  | No | 599(18.79) |  | 588(18.19) |  |
| Pacemaker implantation, n (%) | Yes | 16(18.0) | 0.509 | 11(12.8) | 0.097 |
|  | No | 698(20.85) |  | 671(20.03) |  |
| Mechanical ventilation, n (%) | Yes | 204(49.8) | <0.001 | 213(51.7) | <0.001 |
|  | No | 510(16.85) |  | 469(15.51) |  |
| Coagulase-negative staphylococci, n (%) | Yes | 88(20.0) | 0.647 | 79(19.8) | 0.979 |
|  | No | 626(20.9) |  | 603(19.86) |  |
| Staphylococcus aureus, n (%) | Yes | 142(28.2) | <0.001 | 125(27.7) | <0.001 |
|  | No | 572(19.51) |  | 557(18.67) |  |
| Streptococci, n (%) | Yes | 81(12.5) | <0.001 | 76(10.2) | <0.001 |
|  | No | 633(22.71) |  | 606(22.55) |  |
| Enterococci, n (%) | Yes | 96(17.2) | 0.024 | 72(14.8) | 0.002 |
|  | No | 618(21.47) |  | 610(20.69) |  |
| Streptococcus pneumoniae, n (%) | Yes | 1(6.7) | 0.177 | 2(15.4) | 0.686 |
|  | No | 713(20.84) |  | 680(19.87) |  |
| Anaerobes, n (%) | Yes | 2(9.5) | 0.202 | 3(15.8) | 0.656 |
|  | No | 712(20.85) |  | 679(19.87) |  |
| Gram-negative bacilli, n (%) | Yes | 50(18.9) | 0.443 | 51(19.6) | 0.922 |
|  | No | 664(20.93) |  | 631(19.87) |  |
| Candidiasis / Aspergillosis, n (%) | Yes | 2(66.7) | 0.050 | 6(54.6) | 0.004 |
|  | No | 712(20.74) |  | 676(19.74) |  |
| Readmissions, n (%) | Yes | 152(22.1) | 0.331 | 144(22.8) | 0.041 |
|  | No | 562(20.44) |  | 538(19.19) |  |

T2DM: Type 2 diabetes mellitus. SD: Standard deviation. CCI: Charlson Comorbidity Index. COPD: Chronic obstructive pulmonary disease.
